# Direct transplantation of mesenchymal stem cells into the knee joints of Hartley strain guinea pigs with spontaneous osteoarthritis

**DOI:** 10.1186/ar3735

**Published:** 2012-02-07

**Authors:** Mitsuhiko Sato, Kenzo Uchida, Hideaki Nakajima, Tsuyoshi Miyazaki, Alexander Rodriguez Guerrero, Shuji Watanabe, Sally Roberts, Hisatoshi Baba

**Affiliations:** 1 Department of Orthopaedics and Rehabilitation Medicine, Faculty of Medical Sciences, University of Fukui, Matsuoka-Shimoaizuki 23, Eiheiji, Fukui 910-1193, Japan; 2 Institute for Science & Technology in Medicine, Keele University at the RJAH Orthopaedic Hospital, Oswestry SY10 7AG, UK

## Abstract

**Introduction:**

Mesenchymal stem cells (MSCs) can differentiate into various connective tissue cells. Several techniques have been used for the clinical application of MSCs in articular cartilage repair; however, there are many issues associated with the selection of the scaffold material, including its ability to support cell viability and differentiation and its retention and degradation *in situ*. The application of MSCs via a scaffold also requires a technically demanding surgical procedure. The aim of this study was to test the outcome of intra-articular transplantation of mesenchymal stem cells suspended in hyaluronic acid (HA) in the knee joints of Hartley strain guinea pigs with spontaneous osteoarthritis (OA).

**Methods:**

Commercially available human MSCs were cultured, labeled with carboxyfluorescein diacetate succinimidyl ester (CFDA-SE), suspended in either PBS or HA, and injected into the knee joints of 7-month-old animals. The control animals were injected with either PBS or HA alone. The animals were sacrificed at 1, 3, and 5 weeks post transplantation, the knee joints harvested, and fluorescent microscopic analysis was performed. Histological and immunohistochemical analysis were performed at 5 weeks post transplantation.

**Results:**

At 5 weeks post transplantation, partial cartilage repair was noted in the HA-MSC group but not in the other groups. Examination of CFDA-SE-labeled cells demonstrated migration, differentiation, and proliferation of MSC in the HA-MSC group. There was strong immunostaining for type II collagen around both residual chondrocytes and transplanted MSCs in the OA cartilage.

**Conclusion:**

This scaffold-free and technically undemanding technique appears to result in the regeneration of articular cartilage in the spontaneous OA animal model. Although further examination of the long-term effects of transplantation is necessary, the findings suggest that intra-articular injection of HA-MSC mixture is potentially beneficial for OA.

## Introduction

Osteoarthritis (OA) of the knee joint is characterized pathologically by degeneration of articular cartilage, sclerosis of the subchondral bone, and marginal osteophyte formation, and is characterized clinically by chronic devastating pain and disability in the elderly. OA is a major public health problem and its prevalence is expected to increase dramatically and rapidly over the next 20 years with an increasingly aged population [1]. Although tibial osteotomy and total knee arthroplasty have been pursued in a large number of patients to eliminate joint pain and improve joint function, the majority of patients with knee OA are managed conservatively with medication and/or physiotherapy. Development of less technically demanding but effective therapies for knee OA, such as cell transplantation with or without scaffold enhancement, is therefore desirable.

Mesenchymal stem cells (MSCs) have the capacity to differentiate into a variety of connective tissue cells [2-6]. Several techniques have been used for the clinical application of MSCs in articular cartilage repair [7,8]. In general, the cells are delivered into either the cartilage or bone using a three-dimensional scaffold fixed to the articular defect site. There are many issues associated with the selection of the scaffold material, however, including its ability to support cell viability and differentiation and its retention and degradation *in situ*. Moreover, the application of MSCs via a scaffold usually requires a technically demanding surgical procedure. On the other hand, direct intra-articular injection of MSCs has only been carried out in a limited experimental setting [9,10]. In these animal studies, autologous MSCs - mixed with a dilute solution of sodium hyaluronan (hyaluronic acid (HA)) as a cell binding or cytotactic factor - were directly injected into the knee joint of surgically induced knee OA or focal cartilage defect in certain animal models. The procedure resulted in retardation of the progression of destruction of the degenerative cartilage. Although the injection of MSCs in HA may be the simplest approach clinically, disease progression is rapid in these models, thus making it less amenable to therapeutic intervention [11]. The potential outcomes of this method as a treatment for the slowly progressive process of cartilage degeneration, as commonly occurs in human OA, are still unknown.

The objective of the present study was to determine whether intra-articular injection of MSCs suspended in HA solution into the knee joint enhances the repair of degenerated cartilage in an animal model of spontaneous OA. We used Hartley strain guinea pigs because these animals spontaneously develop degenerative cartilage changes in the knee joint that mimic those of human OA [11-13]. The disease is generally bilaterally symmetrical on the medial tibial plateau in an area unprotected by the meniscus, and the earliest changes can be seen when animals are approximately 3 months old [12,13]. This animal model is more suitable for the evaluation of disease-modifying treatments.

## Materials and methods

### Experimental animals

Seven-month-old male Hartley strain guinea pigs (*n *= 60) weighing 928 ± 4.8 g (mean ± standard deviation) were used. The experimental protocol met the Animal Care Ethics Committee Guidelines for Experimental Studies of Fukui Medical University and Institutional Review Board Guidelines for Stem Cell Research.

### Preparation of human MSCs for *in vivo *tracing

Human MSCs were purchased from Lonza (lot number PT-2501; Lonza, Walkersville, MD, USA). The cells were cultured at 37°C in a humidified atmosphere of 5% CO_2 _in mesenchymal stem cell growth medium (MSCGM™; Lonza). The medium was replaced every 3 days. Seven to 10 days after incubation, the cells reached 75% confluence in the seeded flask and were subcultured. Cells from passages three to five were used for the experiments. For *in vivo *tracing, the MSCs were labeled with carboxyfluorescein diacetate succinimidyl ester (CFDA-SE) (Vybrant CFDA SE Cell Tracer Kit (V-12883); Molecular Probes, Eugene, OR, USA) as described previously [14].

### Intra-articular injection of MSCs

The labeled cells were carefully introduced into a new syringe together with 2 ml HA (molecular weight 8 × 10^5^, Artz^®^; Seikagaku Kogyo, Tokyo, Japan), and the cell suspension containing 7.0 × 10^6 ^labeled MSCs was injected into the medial compartment of the left knee joint of 7-month-old guinea pigs. For this purpose, an 18-gauge needle was inserted posterior to the medial edge of the patellar ligament, through the triangle formed by the epicondyle of the femur, the meniscal/tibial plateau, and the notch formed by their junction.

### Subtyping of animal groups and histological samples

The animals were divided into four treatment groups at random: PBS group (*n *= 15), HA-injected group (*n *= 15), PBS + MSC group (*n *= 15), and HA + MSC group (*n *= 15). The control groups were similar to the test groups with respect to age and weight. In each group, the animals were sacrificed at 1, 3, and 5 weeks post transplantation of cells and/or carrier. The proximal tibia was dissected out and prepared for examination.

### Macroscopic examination

The surface of the distal head of the femur and the tibial plateau were exposed. India ink (2 ml) was injected onto the joint surface using a syringe, and 1 minute later the surfaces were washed with physiological saline. The staining pattern of the cartilage surface was observed macroscopically. The gross findings were classified and scored into six grades according to the assessment suggested by Hayashi and colleagues [15]. The assessment was conducted by two independent examiners (HN, TM), who were blinded to each other's findings and to the treatment group assignment of the animals. Finally, the scores evaluated by these two examiners were averaged to obtain the overall score.

### Histological and immunohistochemical examinations

The proximal tibia was harvested and fixed in 10% buffered formaldehyde solution at 4°C for 48 hours. The samples were snap frozen in liquid nitrogen and then embedded in optimal cutting temperature compound. The frozen blocks were cut into sections 10 μm thick according to the methods described previously [16]. Serial sections 10 μm thick, 20 frontal sections in each knee, were prepared carefully in order to include the severely degenerated area not covered by meniscus. Sections from each animal were used for histological, immunohistochemical, and fluoroscopic analyses.

The sections were stained with H & E and safranin O using standard methods. We assessed the severity of knee OA using the modified Mankin criteria described by Armstrong and colleagues [17], expressed as the OA score. All sections were graded by three independent observers blinded to the treatment group, and the median score was used for statistical analysis.

The tissue sections were also stained with a primary polyclonal antibody, raised in rabbits against type II collagen (dilution 1:200; COSMO BIO, Tokyo, Japan), for 1 hour at room temperature. After rinsing in PBS, the tissue was incubated with biotinylated horse anti-rabbit IgG secondary antibody for 30 minutes at room temperature. Immunohistochemical staining was detected with VECTA STAIN ABC Reagent (Vectastain Elite Kit; Vector Laboratories, Burlingame, CA, USA), followed by staining with 3,3-diaminobenzidine tetrahydrochloride (Dojin Chemicals, Tokyo, Japan). In addition, to evaluate the location of collagen type II relative to the cells, other sections were incubated with the secondary antibody for goat anti-rat Alexa Flour^® ^568-conjugated antibody (1:250; Molecular Probes) for 1 hour at room temperature. These sections were counterstained with the nuclear marker 4',6-diamidino-2-phenylindole.

Cells labeled with CFDA-SE can be visualized by fluorescence microscopy using standard fluorescein filter sets. The approximate excitation and emission peaks of this product after hydrolysis are 492 nm and 517 nm. The labeled cells were quantified in 500 × 500 μm^2 ^areas of tibial frontal section (original magnification, ×100).

### Immunoblot analysis

Immunoblot analysis was carried out with 15% SDS-PAGE. Total protein (80 μg/lane) extracted from the cartilage was transferred onto polyvinylidene difluoride membrane for 70 minutes using a semi-dry blot apparatus. The membrane was washed twice in PBS containing 0.05% Tween 20, blocked by 5% skimmed milk in PBS for 1 hour at room temperature, and then incubated with the collagen type I, collagen type II, or matrix metalloproteinase-13 primary antibody (dilution 1:20; COSMO BIO) overnight at 4°C, followed sequentially by anti-mouse IgG antibody and avidin-biotinylated peroxidase complex (for 30 minutes each). After triple washing in 0.1 M PBS, the membrane was incubated in enhanced chemiluminescence reagent for 1 minute, and then the antibody binding was visualized using a FluorChem™ 8000 System (Alpha Innotech Corporation, San Leandro, CA, USA). The size of each band was normalized to β-actin (lot number IMG-5142A, 1:1,000; Imgenex, San Diego, CA, USA).

### Statistical analysis

Data are expressed as the mean ± standard deviation. Differences between groups were examined for statistical significance using one-way analysis of variance followed by the Bonferoni/Dunn *post-hoc *paired test for comparison between groups. *P *< 0.05 denoted the presence of a significant difference between groups. The above tests were conducted using the Statistical Package for Social Sciences (version 11.0; SPSS Inc., Chicago, IL, USA).

## Results

### Assessment of xenogeneic transplantation of MSCs

**Figure 1:**
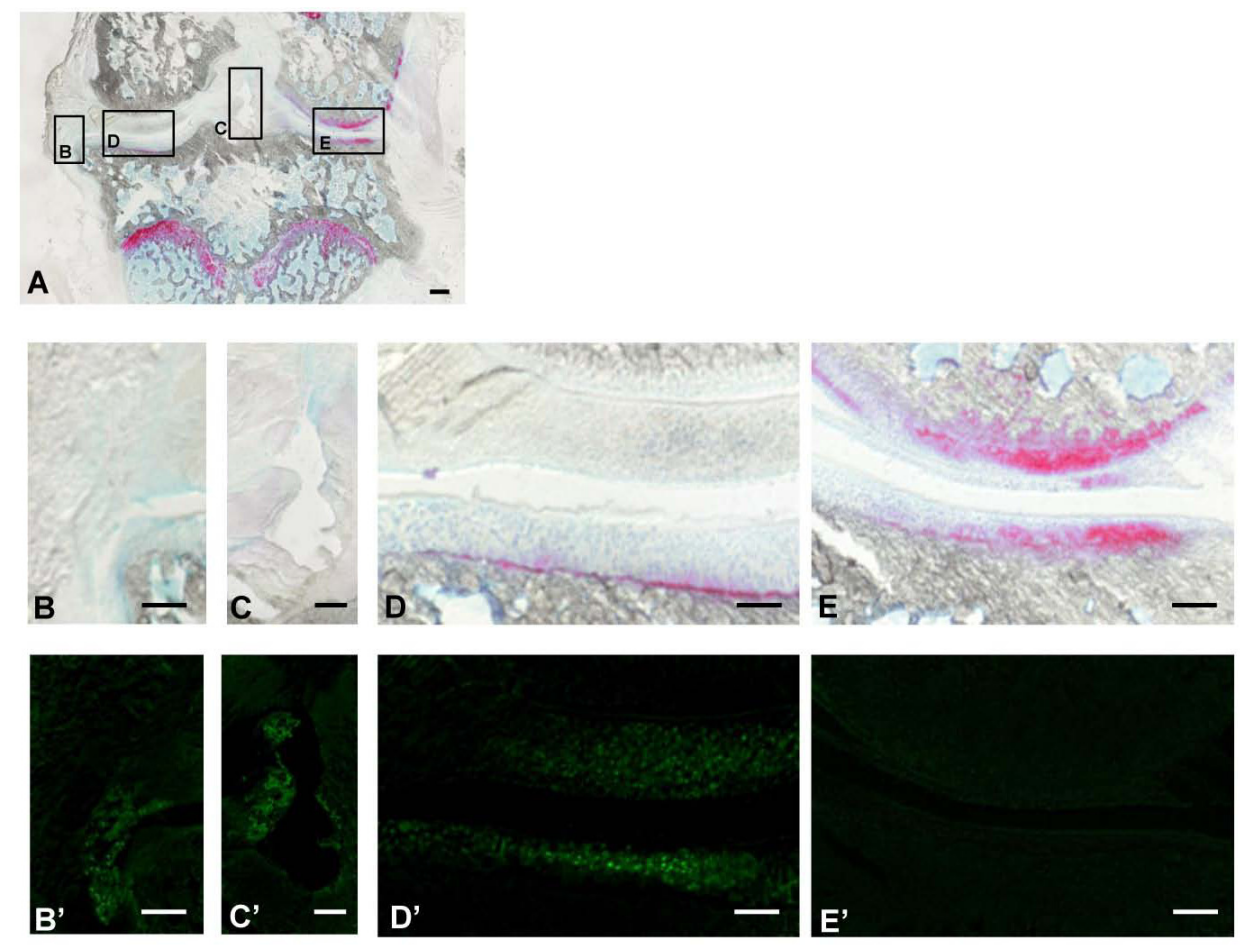
**Xenogeneic transplantation of mesenchymal stem cells**. **(A to E) **Photomicrographs showing frontal sections, 10 μm thick, of the whole knee joint stained with safranin O. **(B' to E') **Fluorescent microscopy for *in vivo *tracing of injected mesenchymal stem cells (MSCs). Bar = 500 μm (A), 200 μm (B to E, B' to E').

Clinically, all guinea pigs tolerated the cell injection, and there was no evidence of local inflammation, joint effusion, or unloading of the joint resulting from the cell treatment. Histologically, the frontal section of the entire knee joint showed no evidence of synovial hyperplasia (Figure 1A, B, C). Fluorescence examination showed labeled MSCs in the fibrillated cartilage at the medial tibial plateau (Figure 1D'). A few labeled cells were also detected in the synovial lining and medial meniscus (Figure 1B', C'), but no such cells were found in the lateral region of the articular cartilage and meniscus (Figure 1E').

### Macroscopic findings

**Figure 2:**
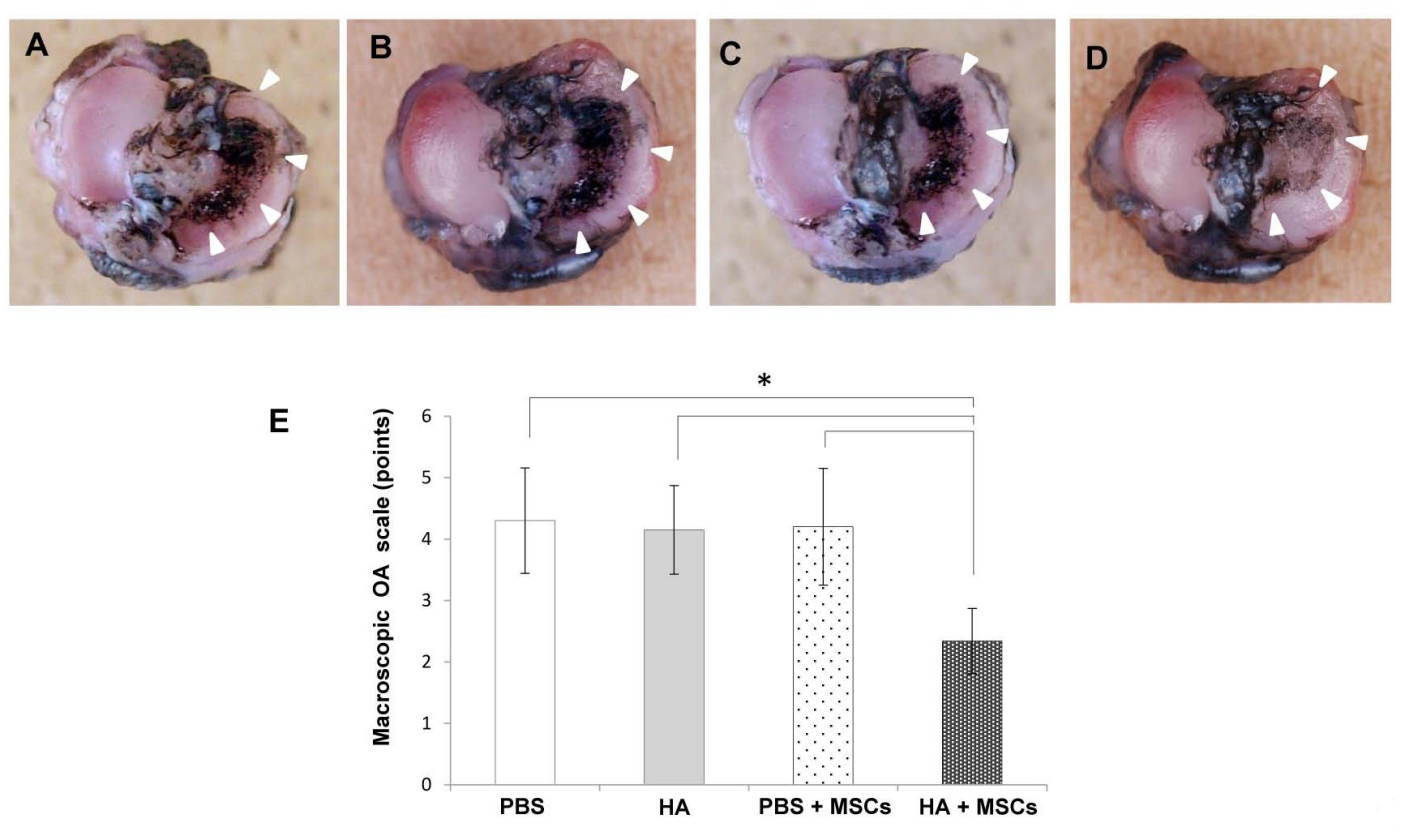
**Macroscopic findings**. Macroscopic photographs (bird's-eye view) of the articular surface of the tibial plateau 5 weeks after injection of each reagent in Hartley strain guinea pigs, stained with India ink: **(A) **PBS group, **(B) **hyaluronic acid (HA) group, **(C) **PBS + mesenchymal stem cell (MSC) group, **(D) **HA + MSC group. Arrowheads: stained lesions. **(E) **Macroscopic osteoarthritis (OA) scores in the same animal groups evaluated 5 weeks after injection. Data are mean ± standard deviation of five guinea pigs in each group. *P < 0.05.

No India ink staining was observed in the lateral tibial plateau of any specimen. In the PBS, HA, and PBS + MSC groups, the surfaces of the articular cartilage over the bare medial tibial plateau (not covered with the meniscus) were rough and strongly stained at 5 weeks post transplantation (Figure 2A, B, C). In the HA + MSC group, the surface of the medial plateau was relatively smooth and the staining intensity was weaker in the same time interval (Figure 2D). The macroscopic OA score was significantly lower in the HA + MSC group than in the other groups (Figure 2E).

### Histological findings

**Figure 3:**
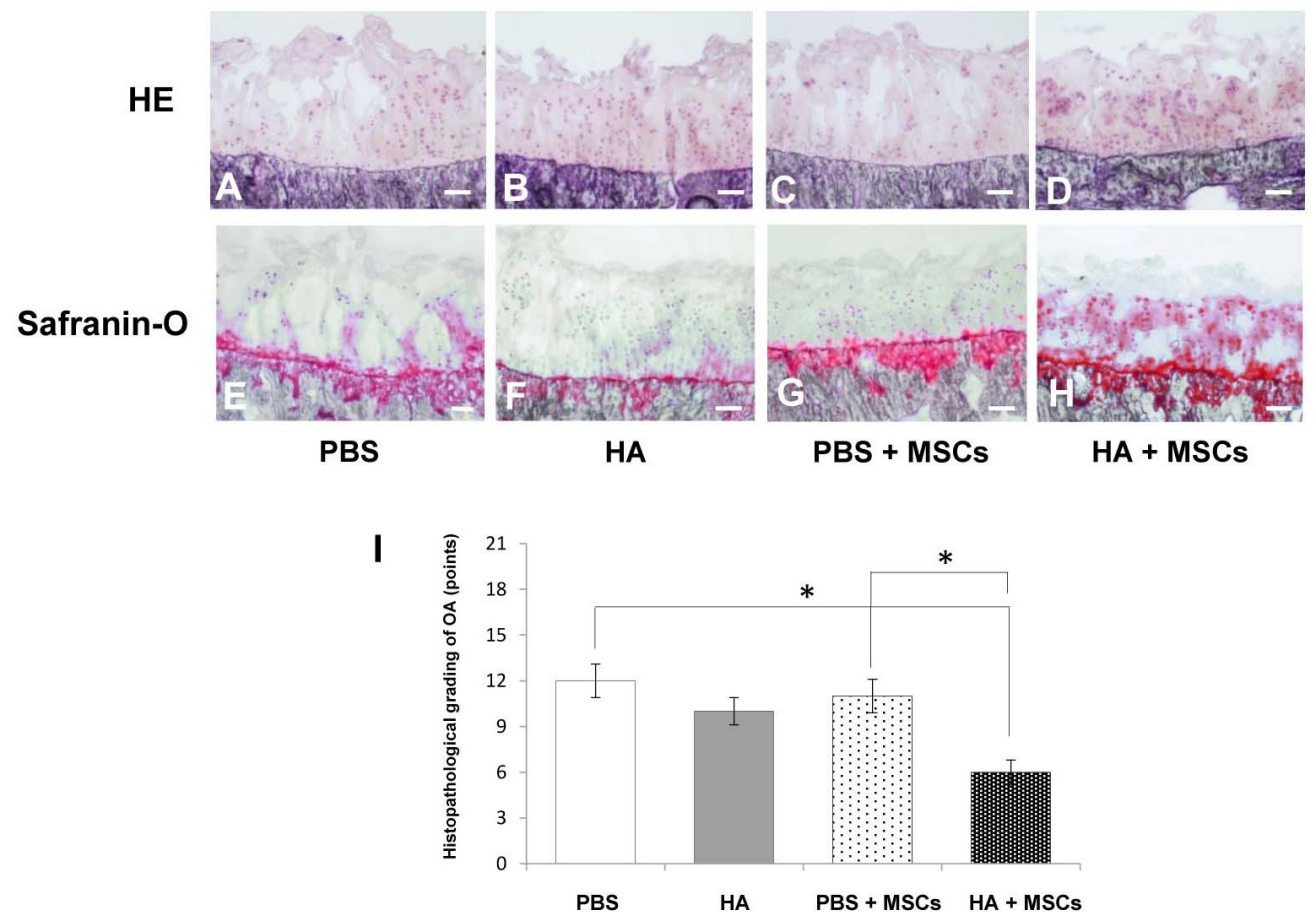
**Histological findings**. Photomicrographs showing serial frontal sections 10 μm thick stained with **(A to D) **H & E and **(E to H) **safranin O. Bar = 100 μm in all sections. **(I) **Histological grade of knee osteoarthritis assessed by modified Mankin criteria 5 weeks after injection, according to the treatment modality. Data are mean ± standard deviation of five guinea pigs in each group. *P < 0.05. HA, hyaluronic acid; MSC, mesenchymal stem cell.

The PBS and PBS + MSC groups showed depletion of chondrocytes (which extended to the transitional zone) and matrix fibrillation (which extended from the transitional zone to the radial zone) (Figure 3A, C). The safranin-O-stained matrix was reduced in all treatment groups with the exception of the HA + MSC group (Figure 3E, G). In the HA group, matrix fibrillation of the articular surface did not extend to the radial zone (Figure 3B) and weak matrix staining and cell depletion were observed (Figure 3F). The HA + MSC group showed large numbers of chondrocytes with cluster formations in the radial layer of the articular cartilage. Furthermore, the matrix around the cell clusters was strongly stained in safranin-O-stained sections at 5 weeks post transplantation (Figure 3D, H).

At 5 weeks post transplantation, significant differences were noted between the OA scores of the PBS and HA + MSC groups, and between the scores of PBS + MSC and HA + MSC groups (Figure 3I).

### Immunohistochemical and immunoblot analyses

**Figure 4:**
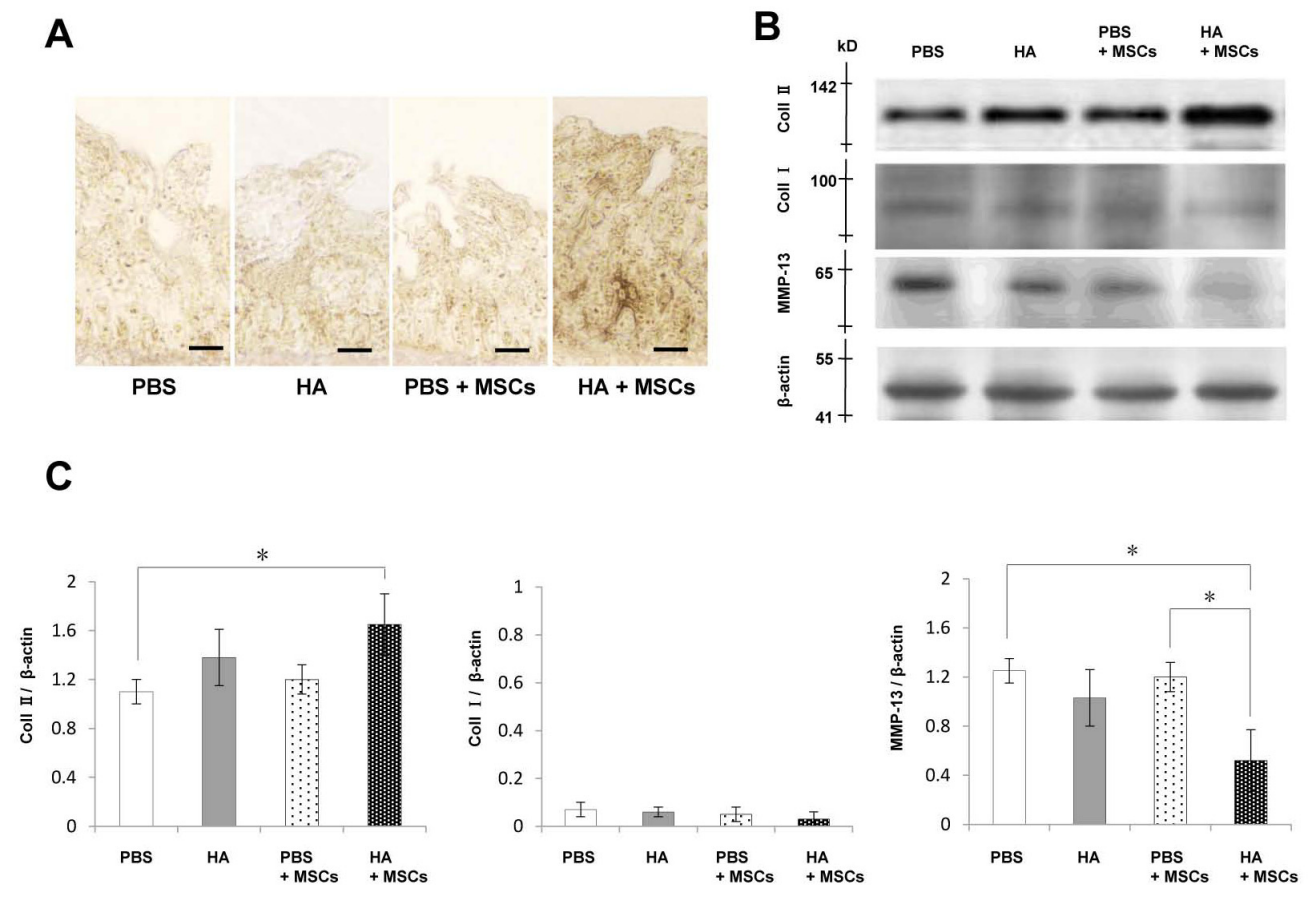
**Immunohistochemical and immunoblot analyses**. Immunohistochemistry, western blotting, and quantification of type II collagen, type I collagen, and matrix metalloproteinase (MMP)-13 expressions at week 5 after injection. **(A) **Immunohistochemical analysis for type II collagen. Bar = 50 μm. **(B) **Western blot analysis of type II collagen, type I collagen, and MMP-13 (pooled samples in each group, *n *= 5). **(C) **Quantitative analysis of the expressions of type II collagen, type I collagen, and MMP-13 relative to the intensity of β-actin. Data are mean ± standard deviation of five guinea pigs in each group. *P < 0.05. HA, hyaluronic acid; MSC, mesenchymal stem cell.

The immunostaining for type II collagen was stronger and more extensive around the chondrocyte-like cells at 5 weeks in the HA + MSC group compared with the other three treatment groups (Figure 4A). Similarly, western blot analysis showed higher levels of type II collagen in the HA + MSC group, relative to the PBS group (Figure 4B). Weak bands of type I collagen were observed in every group, and there were no significant differences between the groups. The bands of matrix metalloproteinase-13 were weaker in the HA + MSC group, relative to the PBS and PBS + MSC groups (Figure 4B). Quantitative analysis confirmed these findings (Figure 4C).

### Fluorescent microscopic findings

**Figure 5:**
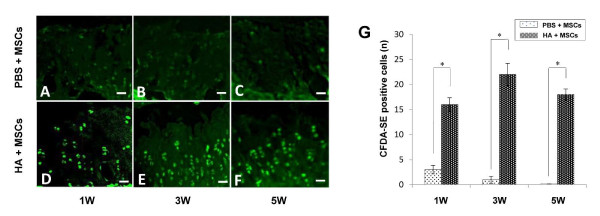
**Fluorescent microscopic findings**. Fluorescent microscopy for *in vivo *tracing of injected mesenchymal stem cells (MSCs) at 1, 3, and 5 weeks. **(A to C) **PBS + MSC group. **(D to F) **Hyaluronic acid (HA) + MSC group. Bar = 50 μm. **(G) **Quantification of the carboxyfluorescein diacetate succinimidyl ester (CFDA-SE)-labeled MSCs. Data are mean ± standard deviation of five guinea pigs in each group. *P < 0.05.

A few CFDA-SE-labeled cells were found within the cartilage at 1 week post transplantation of the PBS + MSC group (Figure 5A). The labeled cells gradually disappeared from the cartilage at 3 and 5 weeks post transplantation (Figure 5B, C). In contrast, the labeled cells appeared both adhered to the surface and scattered within the superficial and transitional layers of the cartilage at 1 week post transplantation in the HA + MSC group (Figure 5D), but they were more frequent in the transitional layer and showed a columnar arrangement at 3 and 5 weeks (Figure 5E, F). The number of labeled cells was higher in the HA + MSC group than in the PBS + MSC group at all the time periods (Figure 5G). These findings suggest that the CFDA-SE-labeled MSCs in HA migrated into the OA cartilage and were associated with the improved metachromasia and histology OA score.

**Figure 6:**
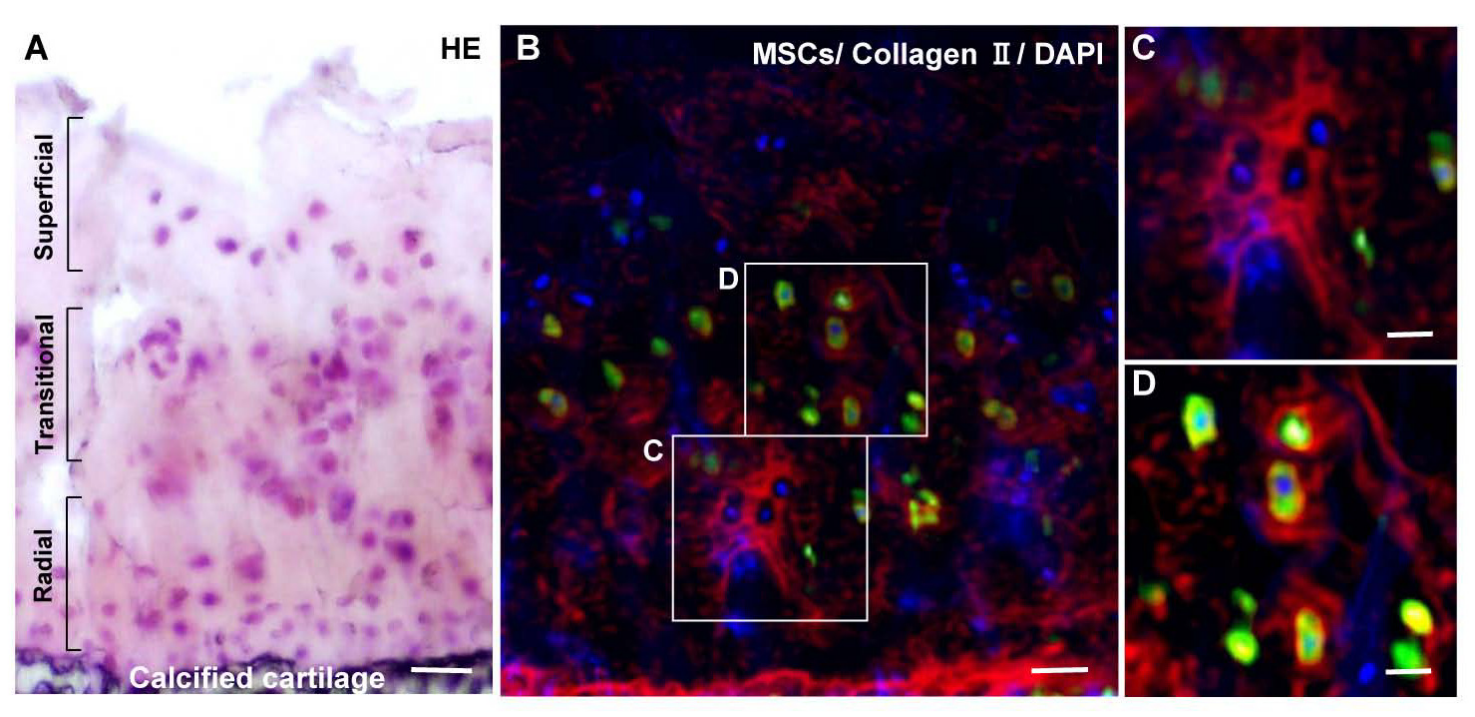
**Double immunofluorescence microscopic findings**. Photomicrographs showing serial sections of articular cartilage 5 weeks after injection in the hyaluronic acid (HA) + mesenchymal stem cell (MSC) group stained with **(A) **H & E and **(B to D) **double immunofluorescence (red, type II collagen; green, MSCs; blue, 4',6-diamidino-2-phenylindole (DAPI)). (B) corresponds to (A). (C) and (D) are magnified views of the boxes marked in (B). Bar = 100 μm (A, B), 50 μm (C, D).

Double immunofluorescence stained sections were evaluated for the distribution of residual chondrocytes, labeled MSCs, and type II collagen in the OA cartilage at 5 weeks post transplantation of HA + MSC injection. Immunostaining for type II collagen was observed in the matrix around residual chondrocytes in the radial layer at 5 weeks (Figure 6B, C), in addition to pericellularly and in the matrix between labeled MSCs in the transitional layer (Figure 6B, D). These findings indicated chondrogenic differentiation of the injected MSCs.

## Discussion

MSCs can be isolated from a variety of mature tissues, and readily expand in culture without losing their multilineage differentiation potential [4,18,19]. Moreover, MSCs secrete a broad spectrum of bioactive molecules, collectively known as trophic factors [20]. Previous reports, however, have demonstrated that MSCs represent only 0.01 to 0.001% of the total nucleated cells within isolated bone marrow aspirates [4] and that the true identity of MSCs is often confused by various isolation and *in vitro *culture methods [21]. In this regard, preparation of large quantities of autologous MSCs for therapeutic use from small experimental animals is sometimes technologically difficult. Several groups reported recently that MSCs produce molecules that are directly involved in the regulation of the immune response and have immunosuppressive properties, which may be particularly important in allogeneic MSC transplantation [22,23]. In addition, human MSCs evade xenogeneic immune reactions and inhibit the inflammatory response in collagen-induced arthritis in mice [24]. For these reasons, we used commercially available human MSCs for xenogeneic MSC transplantation in this study. In the present study, the guinea pigs tolerated the cell injections well, and there was no evidence of local inflammation. Frontal histological section of the whole knee joint showed no evidence of synovial hyperplasia. Fluorescence microscopy also identified the presence of the labeled MSCs in the fibrillated cartilage at the medial tibial plateau. A few labeled cells were also detected in the synovial lining and medial meniscus.

In the present study, we evaluated the feasibility of intra-articular injection of MSCs suspended in HA for the treatment of knee OA. Our results at 5 weeks post transplantation showed histologically confirmed partial repair of the articular cartilage, compared with the three other treatment groups. Immunoblot analysis also showed increasing content of type II collagen and low levels of matrix metalloproteinase-13 in the repaired cartilage. *In vivo *tracing techniques using CFDA-SE labeling and fluorescence microscopy demonstrated that the intra-articularly injected MSCs in HA migrated throughout the osteoarthritic cartilage. Double immunofluorescence analysis also demonstrated strong, although partial, immunostaining of type II collagen around both residual chondrocytes and the injected MSCs after 5 weeks. These results imply intra-articular differentiation of the injected MSCs into chondrocytes and their subsequent proliferation. While HA is known to influence chondrocyte metabolism, the MSCs may produce trophic factors that could also stimulate the residual chondrocytes.

In our animal study, MSCs possibly produced trophic factors for residual chondrocytes, in addition to differentiating into chondrocytes in the presence of HA. While the biochemical conditions necessary for stimulation of chondrogenesis of MSCs *in vitro *have been defined [25], little is known about the differentiation of MSCs into chondrocytes and cartilage regeneration *in vivo*. Several studies investigated chondrogenesis of grafted MSCs within scaffolds in full-thickness cartilage defects in animal models [26,27]. The analyses in these studies showed improved tissue filling and increased matrix staining for type II collagen and proteoglycans 5 to 8 weeks after grafting. The studies' results showed that the transplanted cells secreted type II collagen and contributed to articular cartilage repair. Although simple injection of MSCs was not successful in the repair of cartilage in the present study, MSCs suspended in HA appeared to migrate and proliferate into the transitional layer - and differentiation of these cells into chondrocytes was observed at week 5 after injection. For this treatment group, there was partial repair of cartilage degeneration, both macroscopically and microscopically. The results thus suggest that HA is an important factor in MSC transplantation into the articular cartilage.

There are several advantages for the use of the MSC and HA mixture for intra-articular injection. HA exhibits biological affinity for MSC binding via the transmembrane receptor CD44, which facilitates cell migration through interaction with extracellular HA [28]. HA also increases the chondrogenic activity of MSCs [29]. Another study demonstrated that HA coats the surface of the articular cartilage and also locates among the collagen fibrils and sulfated proteoglycans within the cartilage [30]. Furthermore, *in vitro *experiments indicated that HA alone enhanced the rate of synovial cell migration, and that HA increased chondrocyte migration in the presence of basic fibroblast growth factor [31]. Considered together, our findings and the results of the above studies emphasize the importance of HA in facilitating the migration, adherence, and differentiation of MSCs onto the OA cartilage.

Previous studies used the MSC-HA combination for cartilage repair in surgically induced OA [9,10], but not for spontaneous OA as was carried out in the present study using the Hartley strain guinea pigs. We compared the difference between disease progression of Hartley guinea pigs knees, with and without transection of the anterior cruciate ligament [32]. Their results showed accelerated cartilage degeneration and higher levels of matrix metalloproteinase-13 and IL-1β, which is a key mediator for OA progression, in the anterior cruciate ligament transection model. These results indicated that the progression of OA may differ between surgically induced and spontaneous OA. In this regard, this animal model of spontaneous OA is considered more suitable for the evaluation of disease-modifying OA treatments.

Our study has certain limitations that should be considered when interpreting the results of the experiments. These include the use of nonphysiologic conditions for xenogeneic MSC transplantation, possible deterioration of the repaired cartilage after injection, a lack of ultrastructural analysis such as morphological evaluation by electron microscopy to clarify the *in vivo *chondrogenesis of MSCs in OA cartilage after the injection, a lack of re-examination of optimum timing of injection during OA progression, lack of a MSC population isolated from various types of adult mesenchymal tissues and various issues related to the transplantation of autologous or xenogeneic derived stem cells, and the use of HA only instead of including another compound with molecular weight similar to HA. Further studies are needed that include the above points. Should these issues be resolved satisfactorily, then intra-articular injection of MSCs suspended in HA could be a potentially useful therapeutic intervention for the treatment of spontaneous knee OA.

## Conclusion

The results of the present study suggest that intra-articular injection of MSCs with HA is a potentially beneficial therapy for OA. HA exerts cell-binding and cytotactic effects, thus enhancing the migration and proliferation of MSCs. Although further examination of the long-term effects is necessary, this scaffold-free, minimally invasive treatment seems to retard the progression of spontaneous OA and stimulates the regeneration of articular cartilage.

## Abbreviations

CFDA-SE: carboxyfluorescein diacetate succinimidyl ester; HA: hyaluronic acid; H & E: hematoxylin and eosin; IL: interleukin; MSC: mesenchymal stem cell; OA: osteoarthritis; PBS: phosphate-buffered saline.

## Competing interests

The authors declare that they have no competing interests.

## Authors' contributions

MS carried out most of the experiments, interpreted the data, and drafted the manuscript. KU contributed to study design and conception, analysis of data, and drafting of the manuscript. HN and TM contributed to analysis and interpretation of data and drafting of the manuscript. ARG and SW contributed to acquisition of data and drafting of the manuscript. SR contributed to acquisition of data and critical revision of the manuscript. HB contributed to study conception and critical revision of the manuscript. All authors read and approved the final manuscript.
